# A160 PATIENT SATISFACTION WITH NUTRITIONAL COUNSELLING IN MULTIDISCIPLINARY CARE FOR INFLAMMATORY BOWEL DISEASE

**DOI:** 10.1093/jcag/gwae059.160

**Published:** 2025-02-10

**Authors:** Z Majdzadeh, C Pulver, G Rosenfeld

**Affiliations:** The University of British Columbia Faculty of Medicine, Vancouver, BC, Canada; Western University Schulich School of Medicine and Dentistry - Windsor Campus, Windsor, ON, Canada; The University of British Columbia Faculty of Medicine, Vancouver, BC, Canada

## Abstract

**Background:**

The exact role of diet in inflammatory bowel disease (IBD), including ulcerative colitis (UC) and Crohn’s disease (CD), remains uncertain with no definitive dietary guidelines established. Nutritional interventions in IBD aim to address nutrient deficiencies, alleviate symptoms, and promote long-term remission. Despite the increasing recognition of nutrition in IBD management, evidence on the impact of nutritional counselling in multidisciplinary IBD care centres on patient-reported outcomes remains limited.

**Aims:**

The primary objective of this study is to determine patient satisfaction with nutrition counselling as an adjunct therapy for IBD and explore its potential to improve patient-reported outcomes.

**Methods:**

The IBD Centre of British Columbia (IBDC) is a multi-disciplinary, tertiary care centre in Vancouver, British Columbia that offers nutritional counselling by a registered dietician with experience in managing IBD. Adult patients of the IBDC, who completed at least 3 nutrition counselling sessions for IBD management, were invited to complete an online survey using a 5-point Likert scale that focused on 4 themes: whether the sessions provided new personalized information to patients, their effectiveness in improving patients’ dietary needs, impact on IBD symptoms, and overall patient satisfaction.^1^ Patients who had previously consented to research were invited to participate; ethics was obtained from the Providence Health research ethics board.

**Results:**

A total of 69 patients (44 females, 25 males) completed the survey, with a mean age of 43.440 (+/- 15.48) years, and mean disease duration of 12.6 (+/- 11.23) years. Among these, 41 (59.4%) had CD, and 28 (40.6%) had UC. For the purposes of analysis, responses were categorized as positive, negative or neutral. A significant portion of patients (88.4%) reported that the dietitian sessions successfully managed their IBD related nutritional needs. Additionally, 78.3% incorporated the dietary recommendations into their daily dietary routines. Notably, more than 68% of participants reported that nutritional counselling sessions were effective in improving their symptoms. Overall, patient satisfaction with the dietitian sessions was high, at 76.8%.

**Conclusions:**

High patient satisfaction and symptom relief were reported by patients when surveyed about nutritional counselling as an adjunct therapy in their multidisciplinary IBD care. These findings underscore the importance of integrating personalized dietary interventions into standard IBD care and support the growing recognition that nutrition improves patient-reported outcomes. Future research should explore the impact of nutrition counselling on IBD disease outcomes.

**References:**

1. Craven, Meredith et al. Inflammatory Bowel Disease Patient Experiences with Psychotherapy in the Community. J Clin Psychol Med Settings. 2019;26(2):183-193.

**Funding Agencies:**

None

